# Post-transplant diabetes mellitus: risk factors and outcomes in a 5-year follow-up

**DOI:** 10.3389/fcdhc.2024.1336896

**Published:** 2024-01-30

**Authors:** Matheus Rizzato Rossi, Marilda Mazzali, Marcos Vinicius de Sousa

**Affiliations:** Renal Transplant Research Laboratory, Renal Transplant Unit, Division of Nephrology, Department of Internal Medicine, School of Medical Sciences, University of Campinas - UNICAMP, Campinas, SP, Brazil

**Keywords:** diabetes mellitus, kidney transplantation, immunosuppressive therapy, graft survival, diabetes complication

## Abstract

**Introduction:**

Kidney transplantation is associated with an increased risk of posttransplant diabetes mellitus (PTDM), impacting recipient and graft survivals. The incidence of PTDM ranges from 15% to 30%, with most cases occurring in the first year post-transplant. Some clinical and laboratory characteristics pre- and post-transplant may be associated with a higher PTDM incidence in a more extended follow-up period. This study aimed to analyze the prevalence of PTDM among renal transplant recipients without previous DM diagnosis during a five-year post-transplant follow-up, as well as clinical and laboratory characteristics associated with a higher incidence of PTDM during this period.

**Material and methods:**

Single-center retrospective cohort including kidney transplant recipients older than 18 years with a functioning graft over six months of follow-up between January and December 2018. Exclusion criteria were recipients younger than 18 years at kidney transplantation, previous diabetes mellitus diagnosis, and death with a functioning graft or graft failure within six months post-transplant.

**Results:**

From 117 kidney transplants performed during the period, 71 (60.7%) fulfilled the inclusion criteria, 18 (25.3%) had PTDM diagnosis, and most (n=16, 88.9%) during the 1^st^ year post-transplant. The need for insulin therapy during the hospital stay was significantly higher in the PTDM group (n=11, 61.1% vs. n=14, 26.4%, PTDM vs. non-PTDM). Other PTDM risk factors, such as older age, high body mass index, HLA mismatches, and cytomegalovirus or hepatitis C virus infections, were not associated with PTDM occurrence in this series. During 5-year post-transplant follow-up, the graft function remained stable in both groups.

**Conclusion:**

The accumulated incidence of PTDM in this series was similar to the reported in other studies. The perioperative hyperglycemia with the need for treatment with insulin before hospital discharge was associated with PTDM.

## Introduction

Kidney transplant improves long-term outcomes and survival of chronic kidney disease patients compared with dialysis (1). However, kidney transplant is associated with an increased risk of posttransplant diabetes mellitus (PTDM), which potentially impacts patient and graft survivals and healthcare costs (2–5). PTDM is characterized as a new diabetes mellitus (DM) diagnosed after organ transplantation in patients on a stable maintenance immunosuppressive regimen and absence of acute infection (6). The PTDM incidence ranges from 15% to 30% during the first year, varying according to the study design, diagnostic criteria, follow-up period, risk factors, and immunosuppressive therapy (7). After, the annual incidence of PTDM is around 6% per year, like patients on the waiting list (8). Diagnostic criteria are based on the World Health Organization (WHO) that specify fasting plasma glucose (FPG) of ≥126 mg/dl (7 mmol/L), two-hour glucose after a 75g oral glucose tolerance test (OGTT) ≥ 200 mg/dL (11.1 mmol/L), or symptomatic hyperglycemia with random plasma glucose of ≥ 200 mg/dl (11,1 mmol/L) (9). However, glycated hemoglobin testing (HbA1c) is not recommended for diagnosis during the first three months after a kidney transplant because of the risk of false low levels due to anemia caused by operative blood loss and reduced red cell production secondary to chronic kidney disease (CKD) (9, 10). According to the American Diabetes Association (ADA), if a patient has discordant results from two different tests, the one that presents an abnormality must be repeated (11).

The etiology of PTDM is multifactorial, including general nonmodifiable and modifiable risk factors like the immunocompetent population and some specific conditions associated with the transplantation. PTDM shares many characteristics with type 2 diabetes mellitus (T2DM), such as impaired insulin release and impaired suppression of glucagon release (12). Predisposing PTDM factors common to T2DM include non-Caucasian ethnicity, age over 40, family history of DM, and central obesity (12). These conditions are also associated with the upregulation of pro-inflammatory pathways, with a higher tumor necrosis factor (TNF) expression, aggravating metabolic dysfunction (12). Genetic PTDM susceptibility has been reported, with single nucleotide polymorphisms in genes that encode proteins involved in β-cell apoptosis, ATP-sensitive potassium channels, adiponectin, and leptin (13). The presence of specific human leukocyte antigens (HLA) such as HLA A30, B27, and B42 may also be related (14). Data on HLA antigens associated with PTDM in Brazilian kidney transplant recipients are scarce, although previous studies in this population have identified HLADR13 as a possible PTDM risk factor (4). Central obesity is strongly associated with PTDM, and patients presenting as overweight or obese on the waiting list can be at a higher PTDM risk. The weight gain after transplantation caused by an improvement of a previous uremic condition can also be associated with the PTDM occurrence (12).

Specific risk factors for PTDM include immunosuppressive drugs, acute rejection, cytomegalovirus (CMV) infection, and hepatitis C virus (HCV) infection (3, 7, 14). The incidence of PTDM shows a biphasic curve, with the first peak occurring in the first few months after transplantation, followed by a second peak over 2-3 years post-transplant (12). The first peak is likely associated with the effect of the immunosuppressive treatment in predisposed recipients. The second peak is usually related to recipients’ age and the evolution of classic risk factors for DM combined with specific risk factors related to organ transplantation (12). Recipients of kidney transplantation are exposed to higher doses of steroids during induction of immunosuppression at surgery, followed by oral steroid treatment in tapering doses. Steroids induce peripheral insulin resistance and inhibit the pancreatic insulin production (14). Therefore, in patients at high risk for PTDM, strategies with steroid minimization have been used according to their immunological risk (15, 16). Calcineurin inhibitors (CNI) and mammalian target of rapamycin (mTOR) inhibitors, commonly used for maintenance immunosuppression, are associated with increased insulin resistance, decreased insulin release through direct toxicity to pancreatic beta cells, and impaired insulin-mediated suppression of hepatic glucose production (4, 5, 17–20). Acute rejection episodes can increase the level of insulin antagonist (21), and the drugs used for treating these events can also impact glucose metabolism.

Although the highest incidence of PTDM previously described in the literature occurs in the first year post-transplant, some clinical and laboratory characteristics pre- and post-transplant may also be associated with a higher PTDM incidence in a more extended follow-up period. The primary outcome of this study was to analyze the prevalence of PTDM among renal transplant recipients without previous DM diagnosis during a five-year post-transplant follow-up. Secondary endpoints were clinical and laboratory characteristics associated with a higher incidence of PTDM during this period.

## Methods

Single-center retrospective cohort including kidney transplant recipients older than 18 years with a functioning graft over six months of follow-up between January and December 2018. Recipients younger than 18 years at kidney transplantation and those with previous DM diagnosis were excluded. The cases of death with a functioning graft or graft failure in the six months post-transplant were also excluded. The University of Campinas Ethics Committee approved the study (CAAE 70994523.0.0000.5404).

Patients diagnosed with stage 5 CKD, with an estimated glomerular filtration rate ≤ 10 ml/min/1.73m^2^ or under renal replacement therapy, were evaluated on the kidney transplant waiting list. These patients were investigated for general DM risk factors, such as previous DM diagnosis, current or prior use of hypoglycemic medications or insulin, previous viral infections, and body mass index. Laboratory pre-transplant assessment included FPG, associated with OGTT in some cases. All kidney transplant recipients were submitted to HLA typing and screening of anti-HLA antibodies with solid-phase tests, according to the methodology previously described (22). All kidney transplant recipients presented negative consecutive T and B cell complement-dependent cytotoxicity (CDC) crossmatches at transplant. Induction of immunosuppression consisted of monoclonal interleukin-2 receptor antibody or IV anti-thymocyte globulin 3 mg/kg in cases of standard kidney donors (23) and recipients with panel reactive antibody (PRA) lower than 30% and absence of donor-specific anti-HLA antibodies (DSA). For recipients from expanded criteria deceased donors (23), recipients with PRA > 50% or preformed DSA, and cold ischemia time higher than 24 hours, induction of immunosuppression consisted of pre-transplant IV anti-thymocyte globulin 4.5 to 6 mg/kg. All patients received IV methylprednisolone 500 mg on the day of transplant. Steroids were tapered to IV methylprednisolone 250 mg on the 1^st^ day post-transplant, 125 mg on the 2^nd^ day post-transplant, and oral prednisone 20 mg/day thereafter, with a progressive reduction to achieve a maintenance dose of 5-10mg/day within the 3^rd^ month post-transplant. Furthermore, an ICN (tacrolimus or cyclosporine) was associated with an antiproliferative agent (mycophenolate or azathioprine). The patient’s weight guided the initial dose, then the target level for tacrolimus was 5-10 ng/mL and 800 – 1,000 ng/mL for cyclosporine after 2 hours. Immunosuppression was reduced or withdrawn in opportunistic infections depending on the etiological agent, the patient’s clinical status, and immunological risk.

After transplantation, patients had their blood glucose strictly monitored by measuring capillary blood glucose at least every six hours or whenever necessary. All recipients had the FPG test performed daily during their hospital stay. Patients who presented hyperglycemia during their hospital stay received subcutaneous regular insulin, with dose adjustment according to the measured capillary blood glucose values. In the case of persistent hyperglycemia, treatment with NPH (neutral protamine Hagedorn) insulin was administered in one or two daily subcutaneous applications. After hospital discharge, recipients' clinical and laboratory parameters were evaluated at least monthly, with drug dose adjustments according to their clinical conditions. Among the laboratory parameters routinely evaluated, FPG was performed in all assessments. The HbA1c was performed after the 3^rd^ month post-transplant and repeated every six months. Patients who presented high blood glucose levels in the FPG had a faster reduction in steroid and ICN doses, according to the immunological risk presented. Switching from tacrolimus to cyclosporine was also considered for persistently high values ​​despite other measures. Kidney transplant recipients receiving stable immunosuppressive therapy and more than three months of post-transplant follow-up were diagnosed with PTDM according to the WHO criteria. Patients with an increase in serum creatinine higher than 20% from baseline level or new-onset proteinuria were considered suspected for acute rejection. For these cases, a solid-phase anti-HLA antibody screening was made for detecting donor-specific antibodies (DSA) and a percutaneous graft biopsy, with confirmation of rejection according to Banff 2013 criteria (24). Acute T-cell mediated rejection were treated with IV methylprednisolone 500 mg during three consecutive days or anti-thymocyte globulin 6 mg/kg. Acute antibody-mediated rejection treatment consisted of 5 sessions of plasmapheresis associated with IV intravenous immunoglobulin 2g/kg.

Clinical and laboratory data were retrospectively collected from medical records. Clinical data included the cause of CKD, modality and length of dialysis, donor and receptor characteristics, cold ischemia time, HLA mismatches, hospital length of stay, delayed graft function, and immunosuppressive therapy. Laboratory data included immunological status for hepatitis B virus (HBV), hepatitis C virus (HCV), human immunodeficiency virus (HIV), cytomegalovirus (CMV), mononucleosis, toxoplasmosis, syphilis, and Chagas disease; serum creatinine (mg/dL), proteinuria (urinary protein-to-creatinine ratio, UPCR), fasting plasma glucose (FPG), glycated hemoglobin (HbA1c), and pre-transplant oral glucose tolerance test (OGTT), if available. Furthermore, the incidence of opportunistic infections, including cytomegalovirus, polyomavirus, bacterial and fungal infections, was evaluated. Clinical and laboratory data were collected during hospital discharge and 1^st^, 3^rd^, and every sixth month until five years post-transplant.

For analysis, recipients were divided into two groups, according to the PTDM diagnosis: PTDM and non-PTDM. Statistical analysis was performed using the GraphPad Prisma 9.5.1 program (La Jolla CA, USA), with an unpaired t-test for parametric continuous variables and a Chi-square test for categorical variables. Statistical significance was considered at a value of p < 0.05. PTDM prevalence curve was generated using Kaplan-Meier’s model.

## Results

**Figure 1:**
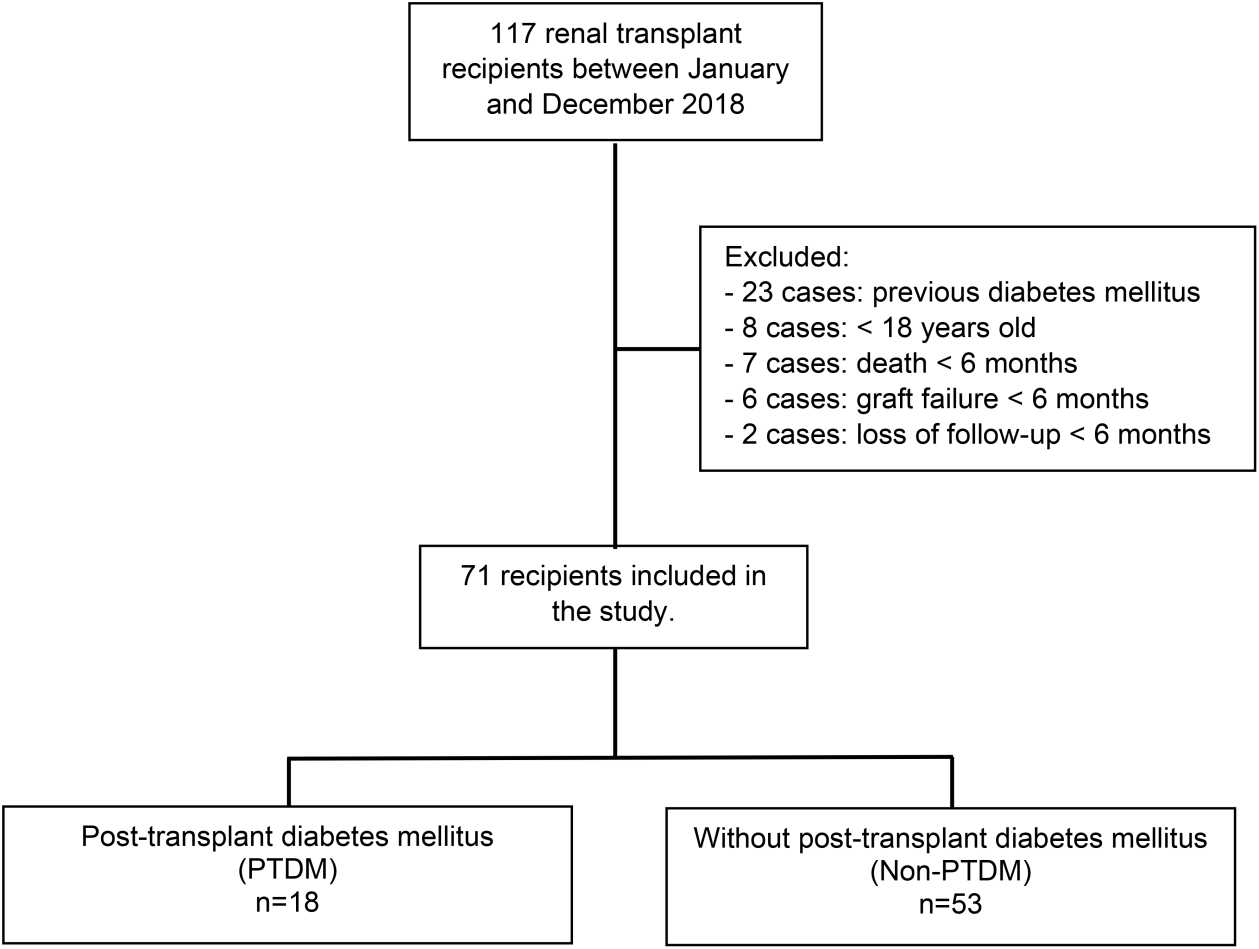
Study population and analyzed groups.

From 117 kidney transplants performed between January and December 2018, 71 (60.7%) fulfilled the inclusion criteria. Forty-six patients were excluded, being 23 (50%) with previous DM diagnosis, 8 (17%) younger than 18 years, 7 (15%) deaths before six months post-transplant, 6 (13%) graft failure before six months post-transplant, and 2 (4%) loss of follow-up. The patients were distributed into two groups according to the PTDM diagnosis (**Figure 1**). Most of the included patients were male (n=42, 59.1%), with a mean age of 46.3 ± 11.7 years. The main CKD etiology was unknown (n=20, 28.3%), followed by hypertensive nephrosclerosis (n=17, 23.9%) and autosomal dominant polycystic kidney disease (n=9, 12.7%). None of the included patients presented chronic HBV infection, 4.2% presented serologic HCV infection, and there was one case of HIV infection. Four (5.6%) recipients presented CMV infection susceptibility (IgG and IgM negatives at transplantation). All the included patients received a kidney from a deceased donor, with a mean age of 42.3 ± 12.0 years and a mean serum creatinine of 1.73 ± 1.77 mg/dL. Thirty-four (47.9%) donors were classified as expanded criteria. Most patients receive induction of immunosuppression with anti-thymocyte globulin (n=68, 95.8%), with doses ranging from 3.0 mg/kg to 6.0 mg/kg, according to the recipient’s immunological risk assessment and donor’s characteristics. In all cases, the initial immunosuppressive therapy includes tacrolimus, associated with an antiproliferative drug (mycophenolate sodium or azathioprine). The mean cold ischemia time was 19.2 ± 4.7 hours, with a delayed graft function (DGF) rate of 35.2% in the general population (**Table 1**).

**Table 1:** General characteristics of kidney transplant recipients according to the post-transplant diabetes mellitus (PTDM) diagnosis.

	General (n=71)	PTDM (n=18)	Non-PTDM (n=53)	p
Recipients				
Age (years)	46.3 ± 11.7	46.5 ± 12.1	45.9 ± 10.7	0.84^×^
Male, n (%)	42 (59.1)	8 (44.4)	34 (64.1)	0.14^¤^
CKD etiology, n (%)				< 0.05^¤^
Unknown	20 (28.2)	7 (38.9)	13 (24.5)	
Hypertensive nephrosclerosis	17 (23.9)	8 (44.4)	9 (17.0)	
ADPKD	9 (12.7)	2 (11.1)	7 (13.2)	
Other	25 (35.2)	1 (5.6)	24 (45.3)	
Hemodialysis pre-transplant, n (%)	67 (94.4)	17 (94.4)	50 (94.3)	0.99^¤^
Time on dialysis (months)	40.6 (5.7–271.8)	35.2 (7.2-67.7)	42.2 (5.7-271.8)	0.90^×^
HBV infection, n (%)	0 (0.0)	0 (0.0)	0 (0.0)	
HCV infection, n (%)	3 (4.2)	1 (5.5)	2 (3.8)	
HIV infection, n (%)	1 (1.4)	1 (5.5)	0 (0.0	
CMV infection susceptibility, n (%)	4 (5.6)	0 (0.0)	4 (7.5)	
Transfusions pre-transplant, n (%)	31 (43.7)	8 (44.4)	23 (43.4)	0.94^¤^
Women with previous pregnancies, n (%)	19 (65.5)	8 (80.0)	11 (57.9)	0.67^¤^
HLA ABDR Mismatches	3.6 ± 0.9	3.5 ± 0.9	3.8 ± 0.9	0.23^×^
Preformed DSA, n (%)	5 (7.0)	0 (0.0)	5 (9.4)	
Donors				
Deceased donors, n (%)	71 (100.0)	18 (100.0)	53 (100.0)	
Age (years)	42.3 ± 12.0	43.3 ± 11.7	39.6 ± 12.8	0.28^×^
Male, n (%)	37 (53.6)	8 (44.4)	29 (54.7)	0.53^¤^
Expanded criteria donors (%)	34 (47.9)	5 (27.8)	29 (54.7)	0.05^¤^
Serum creatinine (mg/dl)	1.73 ± 1.77	1.88 ± 1.84	1.29 ± 1.53	0.22^×^
Transplantation				
Induction of immunosuppression, n (%)				1.00^¤^
Anti-thymocyte globulin	68 (95.8)	17 (94.4)	51 (96.2)	
IL-2 receptor antibody	3 (4.2)	1 (5.6)	2 (3.8)	
Anti-thymocyte dose, n (%)				0.71^¤^
3.0 mg/kg	25 (36.8)	8 (47.0)	17 (33.4)	
4.5 mg/kg	27 (39.7)	5 (29.4)	22 (43.1)	
≥ 6 mg/kg	16 (23.5)	4 (23.6)	12 (23.5)	
Initial immunosuppressive therapy, n (%)				
Tacrolimo	71 (100.0)	18 (100.0)	53 (100.0)	
Mycophenolate sodium	59 (83.1)	15 (83.3)	44 (83.0)	
Azathioprine	12 (16.9)	3 (16.7)	9 (17.0)	
Cold ischemia (hours)	19.2 ± 4.7	19.3 ± 4.7	19.0 ± 5.7	0.84^×^
DGF, n (%)	25 (35.2)	6 (33.3)	19 (35.8)	0.85^¤^
Tacrolimus blood level (ng/mL)				
at hospital discharge (ng/mL)	8.2 ± 4.2	8.0 ± 2.6	8.2 ± 4.7	0.86^×^
1^st^ month	7.4 ± 2.4	7.9 ± 2.8	7.3 ± 2.3	0.37^×^
3^rd^ month	7.1 ± 1.9	6.7 ± 1.3	7.3 ± 2.0	0.24^×^
6^th^ month	6.8 ± 2.0	6.1 ± 1.9	7.0 ± 2.1	0.11^×^
1 year	6.2 ± 1.5	6.3 ± 1.4	6.2 ± 1.6	0.81^×^
5 years	5.5 ± 2.0	4.9 ± 1,4	5.7 ± 2.1	0.13^×^
Time to hospital discharge (days)	12.4 ± 5.7	12.5 ± 6.0	12.1 ± 4.8	0.77^×^
BMI at hospital discharge (kg/m²)	26.0 ± 4.9	25.7 ± 5.0	27.0 ± 4.5	0.30^×^
BMI after 5 years (kg/m²)	27.4 ± 5.7	29.2 ± 6.4	26.9 ± 5.5	0.15^×^
Insulin use before hospital discharge, n (%)	25 (35.2)	11 (61.1)	14 (26.4)	< 0.05^¤^
Acute rejection treated with steroids, n (%)	12 (16.9)	3 (16.6)	9 (16.9)	>0.99^¤^
Infection during the 1^st^ year, n (%)				
Bacteria	17 (23.9)	3 (16.7)	14 (26.4)	0.53^¤^
CMV	15 (21.1)	5 (27.8)	10 (18.9)	0.64^¤^
Polyomavirus	9 (12.7)	2 (11.1)	7 (13.2)	1.00^¤^

PTDM, post-transplant diabetes mellitus; Non-PTDM, without post-transplant diabetes mellitus; n, number; CKD, chronic kidney disease; ADPKD, autosomal dominant polycystic kidney disease; HBV, hepatitis B virus; HCV, hepatitis C virus; HIV, human immunodeficiency virus; CMV, cytomegalovirus; DSA, donor-specific anti-HLA antibody; IL-2, interleukin-2; DGF, delayed graft function, BMI, body mass index.

^×^Unpaired t-test. ^¤^Chi-square test.

**Figure 2:**
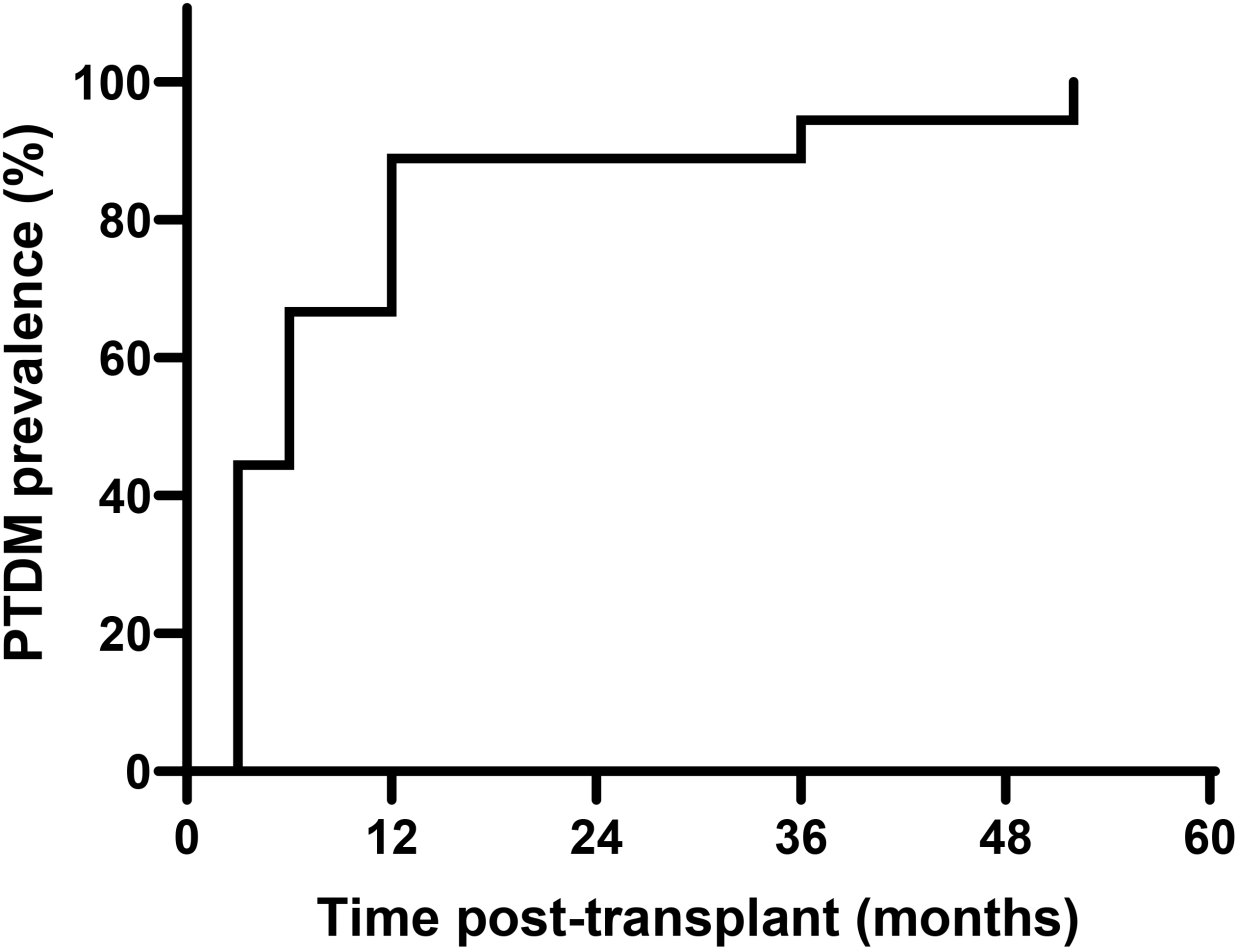
Kaplan-Meier curve of time post-transplant for PTDM occurrence.

Eighteen (25.3%) patients presented PTDM diagnosis, most (n=16, 88.9%) during the first year post-transplant, with half of these cases diagnosed during the first three months post-transplant (**Figure 2**). Most patients of the PTDM group presented CKD secondary to hypertensive nephrosclerosis (n=8, 44.4%), while the mean CKD etiology of the non-PTDM group was unknown (n=13, 24.5%). Hemodialysis was the main previous kidney replacement therapy in both groups. The donors' characteristics and the immunosuppressive treatment were similar between the groups. There was no difference between the groups in the cold ischemia time and DGF rate. There was no difference between the groups in the tacrolimus blood level and the body mass index (BMI) at hospital discharge and throughout follow-up. The need for insulin therapy during the hospital stay was significantly higher in the PTDM group (n=11, 61.1% vs. n=14, 26.4%, PTDM vs. non-PTDM, p<0.05). There was no significant difference between the groups’ occurrence of bacterial, CMV, or Polyomavirus infection rates during the first year post-transplant. At 5-year follow-up, the graft function remained stable in both groups, without significant differences in the serum creatinine or the UPCR compared to the initial values. There were five cases of death with a functioning graft, without significant difference between the groups (n=1, 5.5% vs. n=4, 7.5%, PTDM vs. non-PTDM, p=0.99). The incidence of graft failure was similar between the groups (n=2, 11.1% vs. n=4, 7.5%, PTDM vs. non-PTDM, p=0.63).

## Discussion

In this series, the accumulated incidence within five years of posttransplant follow-up was 25.3%. As previously described, the prevalence of PTDM is variable, with values ranging from 10% to 74%, according to region, population characteristics, and diagnostic criteria (25). A multicenter observational study in South Korea found an incidence of 11.8% within the first year post-transplant (26), while another trial from Slovak Republic showed an incidence of 38,3% in the same period (27). Considering the time post-transplant for PTDM diagnosis, most reported cases occurred during the first year post-transplant, which is probably related to high doses of immunosuppression in this period (8, 28). In this series, most PTDM cases occurred within the first 12 months, supporting previous studies.

All patients received induction and maintenance of immunosuppression according to the protocol at the center. There was no difference in doses of thymoglobulin between the groups. The CNI drugs, especially tacrolimus, play an essential role in the PTDM pathophysiology, increasing insulin resistance and decreasing insulin release (19). A previous study from Saudi Arabia showed that patients with tacrolimus trough level >10 ng/mL during the first three months after the procedure were at higher risk of PTDM, especially in the elderly or overweight recipients (29). In this series, however, the mean tacrolimus blood level was lower than 10 ng/mL throughout follow-up in both groups. Most patients showed a low immunological risk, with less than 10% having preformed DSA, none in the PTDM group. This could have affected the lower dose of immunosuppressive medications used in this series. The current recommended target trough level of tacrolimus for patients with low immunological risk is lower than that used previously, which potentially explains these findings (26). Although the high dose of steroids used to treat acute rejection may be associated with the development of PTDM, there was no difference in the number of acute rejection cases treated in this series.

Other traditional PTDM risk factors, such as age, gender of receptor and donor, ethnicity, BMI, HLA mismatches, acute rejection, and CMV or HCV infection, were not associated with PTDM in this series. One of the reasons that may justify such findings is the relatively small PTDM group. In this series, the need for insulin before hospital discharge was associated with PTDM occurrence. According to the literature, perioperative hyperglycemia is associated with the development of PTDM, mainly when treatment with insulin is required. In a previous retrospective study including 377 kidney transplant recipients, the requirement of insulin therapy during hospitalization posttransplant was associated with a 4-fold increase in PTDM, with 30% of patients treated with insulin before hospital discharge developing PTDM, while 18% of patients who had hyperglycemia without insulin treatment during this period presented this diagnosis (30).

The PTDM management after hospital discharge include oral hypoglycemic drugs alone or associated with insulin (25). Some authors consider metformin as first-line therapy for PTDM (31). However, this drug must be used carefully, especially in patients with impaired renal function, due to the risk of side effects occurrence, such as lactic acidosis (32). Sulfonylureas are also usually prescribed for PTDM, with scarce data on its efficacy and safety (25). New drugs are under study, such as Dipeptidyl-peptidase-4 (DPP-4) inhibitors, which are considered well tolerated, efficacious, and safe in stable kidney transplant recipients with PTDM (7). Observational studies suggest that sodium-glucose cotransporter-2 (SGLT2) inhibitors can be used in this population, with benefits beyond glycemic control (33, 34). In this cohort, the classes of hypoglycemic drugs used were metformin, sulfonylureas, and insulin. One reason for this choice is its availability on the Brazilian public health system, and the higher costs of other drugs.

This study has some limitations, which suggest a cautious interpretation of the data. First, the included patients were from a single hospital in Southeast Brazil, with regional population and dietetics characteristics. Furthermore, each transplant center has its characteristics, such as immunosuppression protocols, and these variations may affect the incidence of PTDM. The study’s retrospective design could also impact the results because of the lack of some information and the possible inclusion of patients with undiagnosed DM before transplantation. Finally, PTDM incidence was lower than other multicenter studies despite the maintenance of steroid therapy during follow-up, which may interfere with statistical strength. However, the study provides valuable information about the effects of perioperative hyperglycemia on PTDM incidence on the long-term post-transplant follow-up.

## Conclusions

PTDM is a condition with a potential impact on the patient and graft survival. In this series, the accumulated incidence within a five-year post-transplant follow-up was 25.3%, most cases occurring during the first year. This can be associated with high doses of immunosuppressive drugs in this period, especially steroids and tacrolimus. Other well-established risk factors for PTDM, such as older age, donor’s and recipient’s age, high BMI, HLA mismatches, and CMV or HCV infections, were not associated with PTDM in this series. The perioperative hyperglycemia with the need for insulin therapy before hospital discharge was associated with PTDM diagnosis.

## Data availability statement

The raw data supporting the conclusions of this article will be made available by the authors, without undue reservation.

## Ethics statement

The studies involving humans were approved by Comitê de Ética em Pesquisa da UNICAMP (CEP-UNICAMP). The studies were conducted in accordance with the local legislation and institutional requirements. The participants provided their written informed consent to participate in this study.

## Author contributions

MR: Data curation, Investigation, Writing – original draft. MM: Conceptualization, Formal analysis, Methodology, Supervision, Writing – review & editing. MS: Conceptualization, Formal analysis, Methodology, Supervision, Writing – original draft, Writing – review & editing.
